# Exploring communities’ and health workers’ perceptions of indicators and drivers of malaria decline in Malindi, Kenya

**Published:** 2017-12-01

**Authors:** Lydiah W. Kibe, Annette Habluetzel, John K. Gachigi, Anne W. Kamau, Charles M. Mbogo

**Affiliations:** 1 Vector Biology Unit, KEMRI - Wellcome Trust Research Program, Kilifi, Kenya; 2 School of Pharmacy, University of Camerino, Camerino, Italy; 3 Social Protection Secretariat, Ministry of East African Community, Labour and Social Protection, Nairobi, Kenya; 4 Institute for Development Studies, University of Nairobi, Nairobi, Kenya

## Abstract

**Background:**

Since 2000, a decrease in malaria burden has been observed in most endemic countries. Declining infection rates and disease burden and reduction in asymptomatic carriers are the outcome of improved quality of care and related health system factors. These include improved case management through better diagnosis, implementation of highly effective antimalarial drugs and increased use of bednets. We studied communities’ and health workers’ perceptions of indicators and drivers in the context of decreasing malaria transmission in Malindi, Kenya.

**Materials and methods:**

A variety of qualitative methods that included participatory rural appraisal (PRA) tools such as community river of life and trend lines, focus group discussions (FGDs) and key informant interviews were used. Studies took place between November 2013 and April 2014.

**Results:**

Providing residents with bednets contributed to malaria reduction, and increasing community awareness on the causes and symptoms of malaria and improved malaria treatment were also perceived to contribute to the decline of malaria. The study identified three perceived drivers to the reported decline in malaria: a) community health workers’ enhanced awareness creation towards household owners regarding malaria-related activities through visitations and awareness sessions, b) Women involvement in Savings Internal Lending Community was perceived to have increased their financial base, thereby improving their decision-making power towards the care of their sick child(ren), c) Non Governmental Organizations (NGOs) and partners played a promoter part in health and general economic development initiatives.

**Conclusions:**

To achieve the goal of malaria elimination, collaboration between governmental and NGOs will be crucial when improving the financial base of women and enhancing participation of community health workers.

## 1 Introduction

Since 2000, a decrease in the malaria burden has been observed in many countries in all World Health Organization (WHO) regions. According to the WHO World Malaria Report 2016, the expansion of malaria control interventions has helped to reduce malaria cases [1]. The WHO document reports that between 2010 and 2015, malaria mortality rates in Africa have fallen by 31% among all age groups, and by 35% among children under five [2]. Specifically, between 2000 and 2010 a reduction of up to 50% of deaths has been reported from several high burden African countries [3], including Eritrea, Rwanda, Zanzibar [4], Pemba, Tanzania mainland [5], Kenya [6] and Zambia [7]. The declining infection rates as well as reduction in the prevalence of asymptomatic carriers are a consequence of improved quality of care, including improved case management related to enhanced diagnostics and implementation of highly effective antimalarial drugs. Large-scale investments in intervention programmes specifically aimed at achieving high coverage of Long Lasting Insecticide-treated Nets (LLINs), campaigns of indoor residual spraying (IRS) and implementation of intermittent presumptive treatment (IPT) in vulnerable groups, have further significantly reduced the malaria burden [4,8].

Other factors not related to interventions have been reported that potentially have an impact on mosquito vectors and vectorial capacity and may have led to a reduction in the numbers of infected individuals. These factors include urbanisation, changes in agricultural practices and land use, and economic development resulting in improved house construction [4]. Climatic changes resulting in altered rainfall patterns, mean relative humidity and temperature have also impacted on malaria vectors in some areas [9].

Notably, most of the studies reporting on changes in malaria epidemiology have focused on the change in the occurrence of human infections and disease [10,11], vector behaviour and vector densities [12] or climatic changes [9,13]. There is a need to also understand communities’ and health workers’ perceptions of malaria risk and their responses in the context of decreasing malaria transmission. This understanding is critical since it influences their attitude to the use of intervention measures and shapes their behaviour towards malaria prevention and control efforts. Studies have shown that bednet ownership in itself is not synonymous with net utilisation and in some settings low net use has been documented [14]. This paper focuses on communities’ and health workers’ perceptions of indicators of malaria decline and drivers facilitating malaria control in a low transmission area in coastal Kenya.

## 2 Materials and methods

### 2.1 Study area

The study was carried out in four villages situated along the river Sabaki in Kilifi County of Coast Region, Kenya. These villages (Jilore, Marekebuni, Malimo, Mikuyuni), are typically rural and situated 40-60 km west of the Indian Ocean on the Nyika plateau. The Nyika plateau is characterised by a hot and dry climate for most of the year with low fertility brown sandy soils, low grassland and thorny bush. Annual rainfall is 500-700 mm and the area is sparsely populated. Most of the people living in these villages (70%) belong the Mijikenda ethnic group. They derive their livelihoods mostly from subsistence farming, fishing and tourism. Homesteads are clustered along the river Sabaki. The river provides residents with water for farming, animal and domestic uses. A detailed description of the study area has previously been published [15,16].

The main malaria vectors in this area are *Anopheles gambiae s.s., An. arabiensis, An. merus* and *An. funestus* [16] and man-made breeding sites have been estimated to be responsible for more than 90% of the mosquito populations [17]. In 2016, the estimated entomological inoculation rate (EIR) was very low averaging 2.3 (range 0.8 to 3.7) infective bites per person per year compared to an annual average EIR of 24.5 twenty years ago [16]. This corresponds to a reduction of more than 90% in malaria transmission over a period of two decades.

### 2.2 Study design and population

The study employed a descriptive cross-sectional study design. Field data collection was carried out over a period of six months, between November 2013 and April 2014 with intervals of between two weeks or one month to allow consolidation and synthesis of data. The study employed a mix of qualitative methodologies with group discussions and key informant interviews as described below and in Tables 1 and 2. A qualitative approach was deemed most appropriate since there was very little existing research on community perception and understanding of factors contributing to the decline in malaria.

**Table 1:** Summary of methods employed based on respondent characteristics and information sought.

Method	Group composition	Information sought
Community river of life	Mixed groups: Old and young; both men and women	Interventions introduced and their impact; incidences and their effects, timelines of occurrences of events
Trend lines	Mixed groups: Old and young; both men and women	Trends on mosquitoes, malaria, mosquito control measures, rainfall pattern, health care, education, women empowerment, social economic status
Focus group discussions	Separate groups: Old men, old women, young men, young women	Common illnesses in the community (past and present); malaria awareness: development partners and their roles, CHWs roles
Key informant interviews	Traditional healers, health care workers, clinicians, laboratory technologist, community development officers	Malaria treatment, activities of TH, use of health care facilities, role of development partners, CHWs participation and challenges

**Table 2:** Summary of methods and numbers of participants involved and frequency of events.

	Villages				
Method	Jilore	Malimo	Marekebuni	Mikuyuni	
Community River of Life					4*
Male	8	11	8	8	
Female	8	10	8	8	
Trend Lines					4
Male	13	11	8	8	
Female	7	7	6	7	
Focus Group Discussions					16
Male (≤35 yrs)	8	10	9	9	
Female (≤35 yrs)	7	7	8	8	
Male (>35 yrs)	9	9	6	8	
Female (>35 yrs)	8	8	8	7	
Key Informant Interviews					16
Male	2	2	2	2	
Female	2	2	2	2	

* Number of events conducted for each method.

Purposive and cluster sampling techniques were used to select the study respondents. In the first instance, the four study villages were identified. Respondents for the study were then purposively selected from these villages using pre -set criteria, namely age (18-70 years), gender, (50% representation of both genders in all age groups) and residency. A chart was developed showing the categories of individuals and the numbers required per age and sex. Selection criteria and the chart were shared with the area chiefs or assistant chiefs of the selected villages to guide in the identification and selection of the respondents. The respondents for key informant interviews were chosen through a snow balling exercise which was based on their involvement in community and malaria-related activities. A total 145 participants took part in the study (129 in group discussions and 16 in key informant interviews). Table 3 provides a summary of the characteristics of the 129 respondents in group discussions.

**Table 3:** Socio-demographic profile of respondents (n=129) involved in group discussions.

Category	Sub - Category	n (%)
Sex	Male	68 (53)
	Female	61 (47)
Age (yrs)	19-35	53 (41)
	36-76	76 (59)
Marital status	Married	106 (83)
	Single	23 (17)
Educational status	Primary	83 (64)
	Secondary	38 (29)
	College/university	9 (7)
Main Occupations	Farmers	101 (78)
	Small traders	20 (16)
	Formal employment	8 (6)

The study team consisted of the principal investigator and 7 other members. Data collection was facilitated by an expert in Participatory Rural Appraisal (PRA) and six Field Assistants (FA) (3 males and 3 females). Before embarking on data collection, the research assistants received a two-day training. The training focused on topics such as ethics in research, use and application of participatory tools in data collection, checking of data for correctness, interviewing techniques and participatory report writing.

### 2.3 Data collection

Data collection methods included Community River of life, Trend lines, Focus Group Discussions and Key Informant Interviews, as follows:

*Community River of Life (CRL):* Respondents were taken through a visualisation of the historical development of their health and environmental issues in relation to malaria. This was done through analogy of a river depicting the chronology of events. They identified the events that the community considered important in its history. These events were either positive (example: provision of mosquito nets, initiation of projects) or negative (‘talking nets’). The positive events were indicated as tributaries while the negative events were indicated as cataracts or waterfalls. Actions that the community took to deal with these events were also discussed and noted. The exercise took about 1.5 hrs. In total, four CRL sessions were held (one in each village). An example of the outcome of a CRL session is shown in Table 4)^.^

**Table 4:** Example of the outcome of a Community River of Life from Marekebuni.

Year	Tributaries	Year	Cataract
1949	CMS church and mosque		
1950	Construction of Marikebuni pry. school	1951	Heavy rainfall-led to crop destruction and increased diseases e.g. malaria, diarrhoea and cholera
1960	Construction of Marikebuni dispensary		
1961	Good harvest in fishing and farming	1961	Flood- led to destruction of crops and houses, diseases such as Malaria, diarrhoea and cholera
1962	Construction of cotton ginnery		
1965	Construction of Masheheni pry. school		
1970	Construction of Galena sec. school		
1974	Constructions of Burangi pry. school		
1979	Burangi rice irrigation scheme project started		
		1980	‘Changilo’ hunger/famine
		1982	Military coup‘mashifta’-somali invaders
1985	Emergence of World Vision (NGO)		
1990	Construction of Majahazini pry. School	1992	‘mitumba’ famine/hunger
1993	Construction of a Kenya Broadcasting Corporation	1997	El nino rains; increased diseases such as malaria, cholera and diarrhoea; destruction of houses and crops
1998	Good farming and plantations- coconuts, mangoes		
2003	Renovation of Marikebuni dispensary		
2005	Construction of Kipangajeni pry. School and emergence of Imani project	2005	Malaria cases and deaths increased
2006	Mass net distribution	2006	‘talking nets’ this led to burning of mosquito nets’
2008	Kipangajeni irrigation project started		
2009	Home management of malaria by Kenya Red Cross society. World Mission work		
2010	Construction of Masheheni-Burangi road; introduction of fish pond project		
2011	Catholic relief services introduced Savings and Internal Leading Community scheme		
2012	Catholic relief services-initiated water project Compassion – Non-Governmental Organization Electricity to the mosque V.C.T room at the dispensary Mass net distribution by Ministry of Health	2012	End of Home Management of Malaria project Jiggers infestation
2013	Construction of Burangi bridge Construction of modern kitchen at Masheheni pry by Catholic Relief Services Shoes by Kenya red cross for prevention of jiggers Burangi irrigation scheme handed over to Government	2013	Bilharzia Jiggers Early pregnancies

*Trend Lines:* Four trend Lines (TL) diagrams were developed (one in each village). The TL depicted the historical patterns of malaria and malaria control in the context of community knowledge. This was done through a matrix with year as unit for a period of ten years and various parameters depicting specific issues in line with the study objectives. The parameters included provision of health services (CHWs, Traditional healers and health facilities), mosquito presence and density, malaria presence and intensity of transmission, gender empowerment and mainstreaming, mosquito control and management. From the trend lines respondents could recognise similarities of certain parameters and understand their relationships. The exercise took 3 hrs.

*Focus Group Discussions (FDGs):* Sixteen FGDs were held with respondents divided according to age and sex per village: young men 35 years and below, young women 35 years and below, older men 36 years and above and older women 36 years and above. Discussions with the older groups preceded the younger groups to allow exploration of their experiences with malaria. The female groups were conducted by a female facilitator while the male groups were conducted by a male facilitator. Six research assistants (3 males and 3 females) helped in note taking and observing the process. The FGDs provided researchers with an opportunity to explore and validate information that was gathered from CRL and TL exercises. Discussions revolved around health problems in the community, role of CHWs in malaria prevention and control, role of government departments and NGOs in malaria prevention and control, sources of information regarding malaria, and effects of SILC on women participation in malaria prevention and control.

*Key Informant Interviews (KII):* A total of 16 KII were conducted in all the 4 study villages using a KII guide. Respondents included 2 health facility in-charges, 1 entomologist, 1 Catholic Relief Services (CRS) officer, 1 Village Community Development Officer, 1 women group leader, 4 traditional healers, 4 CHWs, 1 laboratory technician and 1 public health officer. The information sought included issues on indicators of malaria decline, treatment seeking behaviour, factors contributing to malaria decline, role CHWs in malaria control and prevention, role of development partner’s in malaria control and prevention and innovative ways women are participating in overcoming challenges in malaria prevention and control.

### 2.4 Data management and analysis

Notes from field discussions and debrief sessions were summarised and compiled each day. Pictures of all sketch diagrams were stored in a computer picture folder. Field notes were then organised thematically with NVivo version 10 [18]. The analysis approach was a combination of ethnomethodologies with emphasis on the grounded theory [19], thematic and content analysis [20] guided by the conceptual framework [21,22]. To ensure consistency, the PI and the PRA expert independently generated codes and categories from the data. Later, they agreed upon the codes and categories and formulated the underlying meaning of the different categories of the codes into a theme. Direct quotes arising from the discussion were identified that supported defined themes. The translation of the quotes was done with the help of the field assistants and the two researchers confirmed and checked for accuracy of the translations and interpretation of the quotes presented.

### 2.5 Ethical considerations

Ethical clearance was obtained from the KEMRI ethical review committee (SSC No. 2631). The PI also completed an online ethics refresher course to understand the principles and procedures of conducting ethical human research [23]. Field assistants were trained on ethical procedures. In the field, necessary protocol both at the County department of health and respective local government units was carried out and advanced informed consent was obtained from all respondents.

## 3 Results

Three primary themes were derived from analysing the data set: a) Level of malaria burden as a disease in the study area, b) Indicators and factors contributing to the decline in malaria, and c) key drivers facilitating the decline. Each of these themes is discussed below:

*a) Level of malaria burden as a disease and other health risks in the study area:* The residents perceived malaria as bad and killer disease based on their past experiences with the illness. Nearly all respondents acknowledged to have had a malaria episode in the past. Nonetheless, the respondents noted that malaria severity had declined over the past five years (between 2009 and 2013). Of the diseases mentioned (HIV, cancer, stroke, respiratory disease), malaria was not mentioned as a cause of death in any of the study areas:

“*It is difficult to find a person dying of malaria. Unless one has an underlying cause, malaria alone does not kill nowadays, as it was the case before...”* (KII, HW #1)

“… *I have not seen anyone in my family with malaria in the last four years. Previously, malaria was very common. But nowadays, there are very few cases of malaria”* (FGD, older male respondent, Marekebuni)

“*Cases of patients presenting with malaria were frequent sometimes back (before 2009) but this has changed over the last years, we receive very few patients with malaria”* (KII, HW #3)

“…*unlike before, those whose results are positive are very few. ...most tests are negative. For example, you can test ten patients and only find one patient positive for malaria parasites”.* (KII, HW #4)

*b) Level of Indicators of contributing to malaria decline:* Respondents mentioned various indicators for the decline in malaria as well as factors that contributed to its reduction (Table 5). Overall, increased availability of bednets was perceived as the most important factor, accounting for 20% of responses, followed by increased awareness about malaria (15%). Participation of CHWs in malaria prevention activities at the household level, involvement of NGOs and other development partners in control interventions, and increased numbers of health facilities were considered important. Factors identified by the community residents were similar to those mentioned by the health workers.

**Table 5:** Indicators of and factors contributing to the decline in malaria: frequency of responses by factors / indicators.

Factors and Indicators mentioned	%
Availability of treated mosquito nets in the households	20
Increased awareness about malaria (causes, control and treatment) by women and other members of the community	15
Increased participation of CHWs in malaria prevention activities	10
Increased participation of development partners	10
Increased number of health facilities – reduced walking distance	10
Decreased cases of malaria observed in the household	10
Increased time for other activities e.g. farm work, caring for children	5
Reduced cases of deaths from malaria	5
Improved availability of free anti-malarial drugs in the health facilities	5
Decreased expenditure on treatment	5
Decreased importance of traditional healers in the treatment of malaria.	3
Decreased cases of convulsions due to malaria (yago)	2
Total	100

From FGD sessions it emerged that knowledge about malaria symptoms, treatment of the disease and control of the vector was high among residents of all the villages irrespective of age and gender. Commonly cited sources of malaria information included CHWs’ during home visits (30%), listening to the radio (22%) and informative posters (19%) (Table 6). Other avenues mentioned were health talks during Anti Natal Clinic (ANC) visits, short text messages from the MoH in conjunction with development partners such as Population Service International, urging residents to use bednets continuously and during all seasons. Development partners such as the Kenya Red Cross Society (KRCS) that promoted the Home Management of Malaria (HMM) in Marekebuni between 2009 and 2012 [24] allowed to increase community awareness through CHWs supported by the project. The KEMRI *icipe* malaria programme in 2007 trained community liaison officers in mosquito control in the study villages (except Jilore).

**Table 6:** Sources of information that contributed to the decline in malaria, frequency of responses and information disseminated.

Source of information	Frequency of mention (%)	Information disseminated
Health talks by dispensary staff at the dispensary, Community health workers, Development partners	30	Malaria signs and symptoms, use of preventive measures especially mosquito nets, malaria treatment at the dispensary, adherence to drugs, fever management in children
Radio	22	Use of preventive measures e.g. mosquito nets and visiting the hospital for malaria treatment
Posters displayed at the dispensary	19	Mosquito net use
Chiefs barazas	15	Importance of using mosquito nets.
Telephone short message alerts	7	Use of mosquito nets all the time every season
Television	7	Tailored messages especially during the rainy season encouraging residents to keep the environment clean and to sleep under insecticide treated mosquito nets.

*c) Key drivers facilitating malaria decline:* Among the various factors contributing to the decline in malaria, three key drivers were identified by respondents in the study villages: i) CHWs involvement in health-related activities and malaria at the household level, ii) women empowerment through Saving Internal Lending Community Scheme, and iii) interventions of non-governmental organizations and other partners addressing malaria control and general economic development.

*i) CHWs involvement in health and malaria related activities at the households’ level:* CHWs were present in all the study communities. However, there was a variation in their active involvement and availability for providing malaria-related activities across the four villages. Although TL discussions showed an increase in the number of CHWs in all the 4, there were more CHWs in Marekebuni than in Malimo, Mikuyuni and Jilore. The activities of CHWs were more intense in villages that had on-going projects by development partners. Thus, presence and effective participation of CHWs in malaria control increased alongside activities of development partners. The roles of CHWs and their importance in malaria-related activities as perceived by the community was widely debated during all the discussions. Their role was illustrated as advisory, educative, consultative and evaluative. During FDGs, women (in both age groups) acknowledged the importance of CHWs in encouraging them to visit health facilities when they observe signs of ill health such as fever. In Marekebuni for example, CHWs were more actively involved in malaria management during the Kenya Red Cross home management of malaria project (2009-2013). The CHWs were referred to by the community as ‘*daktari wa dawa moja*” (a doctor of one medicine) since they only had Artemether-lumefantrine (AL) for malaria treatment whereas residents sought them for many other different health problems, which unfortunately they had no medications for.

According to health workers, the reduced incidence of malaria and other illnesses and the increased utilisation of health facilities, is partly due to the health education activities conducted by CHWs:

*“The Red Cross project was very successful here. If the Ministry of Health was to train CHWs to implement community based malaria control, malaria will be history!”* (KII, HW, #4)

The main challenges reported regarding CHW activities were finances to establish community structures, training of the CHWs and maintaining their activities.

*“The Ministry of Health has not been able to support the CHWs with a “CHW kit”* (CRL, older woman, Mikuyuni).

Consequently, it was reported that there was a low participation of CHWs, operating without support from specific donors/sponsors. The drop-out rate was high and hence the number of CHWs who conducted the activities were few:

*“We only see them when they are working with organizations”* (CRL, older woman, Mikuyuni).

*ii) Participation of women in Saving Internal Lending Community (SILC) and its impact on malaria prevention and control:* Generally, the SILC financial intermediation process involves collecting savings, disbursement of loans, making repayments and collect contributions of set amounts towards a social fund. The main objective of the SILC programme is to enable the economically active poor, especially women, to develop their own reliable financial services and to support community self-reliance and resilience [25]. SILC activities were reported more frequently in Marekebuni and Malimo organised by Catholic Relief Services and a few activities in Mikuyuni organized by World Vision. Jilore did not have active stakeholder involvement in SILC. SILC provides the members with opportunities for diversifying their income to provide their families with basic items like food, clothes, shelter, drugs and education for their children. Some expressions from women about SILC:

*“I have used the money from SILC to buy three goats and I paid my son’s secondary school fees from a loan I took from SILC”* (SILC member)

*“You see these motorbikes (pointing at motorbikes packed outside the interview room); most of them are owned by women. The owners (women) employ young men to operate them. By the end of the day, they give the owner an agreed amount”* (FGD, Male, and Malimo)

SILC has empowered women economically, thereby increasing their power in decision making. For example, they can decide on their children’s health issues and seek timely and prompt treatment for illnesses, which is crucial in the case of malaria. Women have been enabled to make decisions on their own health needs, and on those of their children, on the utilisation of family income and investments priorities. Women have raised their educational profile due to their participation in capacity building forums, sharing forums and community meetings organised by SILC. Women commented:

*“We are happy with SILC, we can now have our own money. I do not have to wait for my husband to come and give me money to take my child to the hospital.”* (FGD1, women P6)

*“My child had fever and I did not have money to take her to Malindi Hospital, when I went to SILC, I got money for transport and medicines”* (FGD4, young woman P4)

Organized in SILC groups, women lend money to each other. That way, they develop trust and a sense of responsibility. Members are trained on women’s and children’s rights, book keeping and adhering to the set-out rules:

*“SILC is empowering us in a big way. If you move around in this area, you will see most women having small businesses. When there is no farm work, women are in their businesses”* (FGD3, woman P8)

*“We get soft loans from SILC. This helps us expand or initiate our own businesses”* (KII, women leader 1)

*“The SILC is transforming women’s lives in this area. Look at the women’s faces and you will see they are happy”* (CRL, young woman, Marekebuni)

*iii) Interventions of non-governmental organizations and other partners addressing malaria control and general economic development:* Fifteen organisations were identified and included the KEMRI/*icipe* malaria programme, Aphia Plus, Catholic Relief Services, World Vision, Action Aid, AMREF, Kenya Red Cross, CISP and PSI (Table 7). Also mentioned were government ministries and departments e.g. agriculture, health, education and water and roads. Apart from malaria-related activities, partners such as CISP, World Vision, Redcross CRS worked on cross cutting issues such as provision of water (digging dams, earth pans, boreholes, water tanks), improvement of roads, education and agriculture. Areas like Marekebuni and Malimo had more development partners than Jilore and Mikuyuni (Table 7). This could be explained by their proximity to the town of Malindi. The partners primarily worked with CHWs and dispensary health personnel to provide training, antimalarial drugs, financial, and non-financial materials and supervisory support. Residents appreciated the role played by NGOs and other partners in malaria and mosquito control.

**Table 7:** Development partners working in the field of malaria and mosquito control in the 4 study villages.

Partner	Activities	Area of operation (villages)
KEMRI - *icipe* malaria programme	Entomological research activities: capacity building of PHOs and CHWs in integrated vector management and larval control	All study villages
Kenya Red Cross	Giving mosquito nets to floods displaced families; Home management of malaria; Training CHWs in Malaria management	Malimo, Marekebuni
APHIA Plus	Training of CHWs in malaria management; Strengthening community based health structures through community strategy; Promoting maternal and child health	Malimo, Marekebuni
World Vision	Women empowerment through SILC; Conducts of water and sanitation projects; School health activities	Mikuyuni
Catholic Relief Services (CRS)	Women empowerment through SILC; Conducts water and sanitation projects; Women empowerment activities – women and child rights	Marekebuni,
African Medical Research Foundation (AMREF)	Conducts of School health Program Infrastructure development-building classrooms; Promoting food security through irrigation, digging fish ponds	Marekebuni
PSI	ITNS distribution to under 1 yr. olds & pregnant women and capacity building	All study areas
CISP	Capacity building	Marekebuni
GOK Agencies i.e. CDF, Arid Lands Development project, Ministry of Education, Livestock and Youth affairs	As per the mandate of the ministry touching on environmental management	All study areas

## 4 Discussion

Communities and health workers living along the River Sabaki in Malindi have observed a decline in malaria transmission. This decline was facilitated by increased community awareness about malaria, improved health services and increased ownership of mosquito nets. These 3 drivers have been potentiated by scaled up CHWs performance with providing residents with information on the importance of controlling malaria and encouraging malaria patients to seek treatment at the health facilities, by socio-economic improvement of women through SILC and by various interventions of development partners. Below we discuss the various challenges that were identified which might hinder future malaria elimination efforts in the area.

CHWs performance was scarce in areas that had few or no development partners and this resulted in several key challenges. Firstly, inadequate training and capacity building was found, whereby only a few CHWs had received training during the formation of community units under the Community Health Strategy (CHS) campaign in 2008, or had received refresher training in 2011 in Malimo village. The training was based on a formalised curriculum and focused on general ailments affecting the community. Topics on malaria mainly tackled malaria signs, symptoms and treatment. Information on malaria prevention focused on bednets. However, CHWs are expected to provide information to households on other malaria control and prevention such as larviciding, environmental management, and proper use and care of bednets [24,26,27]. Their performance could be significantly improved through refresher and regular training that appropriately addresses the challenges they face in their daily work. Secondly, insufficient funding to establish, support and supervise CHWs. The study observed that areas with well-established and functional community units (Malimo and Marekebuni) had high participation of CHWs. A community unit is established to support the work of CHWs in training and supervision and is embedded within the health system [28]. Previous studies have emphasised the need for monitoring and supervision of CHWs [29,30]. Kisia *et al.* [24] provided evidence that CHWs can improve malaria diagnosis through close supervision. However, community strategy remains on paper without funding allocated towards its implementation. For the moment, establishing community units within a CHS is dependent on donor support. It requires a substantial amount of money (USD 5000) to establish a functional community unit. With no provisions in the budget, donor projects find it difficult to facilitate the MoH to establish community units. Even in community units that were established with the help of donor projects the support and maintenance of CHWs’ activities declined immediately after the end of donor support. It is therefore important that county governments allocate funds to support the community health strategy if we are keen in achieving the universal health coverage and sustainable development goals by 2030. Thirdly, sustainability of CHWs activities was low. The study distinguishes two categories of CHWs: donor / sponsored / project CHWs and MOH CHWs - not supported by specific donor projects, of which the former was found to be the more active ones (Table 8). Although CHWs are expected to perform activities on a voluntary basis, attrition rate was high after the end of the donor projects. We observed that CHWs that worked with donor funded projects received greater motivation than the non-project CHWs. They received both financial and non-financial incentives. But upon completion of the programmes, the MOH provided minimal support in monitoring and supervision to their activities. Both financial and non-financial incentives have been found to enhance the performance of CHWs [24,31-33]. But the challenge is to establish systems able to continuously sustain the activities of CHWs. Models that have a professionalised approach for community health workers include the Community Health Agents (CHAs) in Tanzania, and the Health Extension Workers (HEW) in Ethiopia. In Tanzania, the CHAs are formally remunerated, receive nine months of preservice training, and dually report to health facility staff and village governments [34]. HEWs in Ethiopia received one year of training, were also remunerated by the government, and reported to nurses or environmental health professionals at nearby health centres [35]. In Kenya, apart from training and remuneration from donors, the model included supervision of CHWs [24]. The county departments of health need to investigate these models for improving sustainability and effectiveness of the Kenyan CHWs.

**Table 8:** Differences between ordinary community health workers (CHWs) and project CHWs.

Project CHWs (sponsored)	Ordinary CHWs
Paid an allowance (transport, lunch) Activities are based on projects objectives	Not paid – services are on voluntary basis Activities are mainly based on MoH* campaigns e.g. polio campaign, sanitation campaigns, net distribution, jigger campaigns
Active during the project period	Active during MoH organized campaigns
(1-3 years or more)	(1–5 days)
Motivated by financial allowances receives, t-shirts, bicycles; recognition by community and MoH	Motivated by their involvement with MoH activities which makes them feel recognized by MoH, the community, presence of MoH staff during dialogue days, expectations of better things to come such as a job, training and other benefits

* MoH: Ministry of Health

In addition, strengthening inter-sectoral collaboration for malaria prevention and control was considered. The study identified several development partners working in these study areas (Table 7). These partners were involved not only in malaria-related activities, but also in overall social and economic development of the area and the communities perceived this to contribute to malaria reduction. These activities included road construction, women empowerment programmes, provision of water through supplying storage tanks, sinking boreholes and earth pans. There were a few partners that participated in malaria-related activities such as formation of community units, training CHWs, maintaining their activities and providing them with support supervision. Training of health facility staff, provision of bednets during emergencies such as floods, sampling mosquitoes, and provision of antimalarial drugs to the health facilities were also important activities contributing to the decline in malaria since they complemented the activities of the MoH. This means that the role of development partners in social and economic development of the people is important to facilitate reductions in malaria. Sanders *et al.* [36] described the importance of public private partnerships in malaria elimination in Malaysia. The challenges of relying on development partners include short life of the donor projects and insufficient coordination of partners’ activities in the field. Thus, discontinuation of the activities, especially those carried out by CHWs, is an important drawback. The final challenge pertains to information and dissemination activities regarding malaria control. The identified information dissemination channels (Table 6) showed that the main message delivered was on bednets. Consequently, there was only one out of fifteen (1/15) stakeholders involved in larval source management (Table 9). Efforts should be made to include messages on the other malaria prevention and control methods. This would provide residents with a variety of methods to choose from and serve as a reminder on their uses.

**Table 9:** Community based malaria control activities and the number of organisations involved.

Activities conducted	Organizations involved
Bednet distribution / use	9
Clean environment	6
Training health staff in malaria prevention and treatment	5
Malaria treatment	2
Strengthening community based health structures through community strategy	2
Larval source management	1

On the other hand, SILC provides good social networks that have not been fully utilised for malaria control and prevention. The group can become an avenue for passing relevant messages and information to women, especially during their meetings. The messages can be passed by CHWs who are also SILC members. Such messages could include bednet use and care, removing standing water to eliminate mosquito breeding places as well as seeking prompt treatment when they signs of fever are being detected.

## 5 Conclusions

We investigated the perceptions of communities and health workers in an area of reported malaria decline in Malindi, Kenya. Both the communities and health workers observed a decline in malaria based on several indicators. Important drivers that influenced this decline are community health workers’ involvement in awareness creation on malaria prevention and control, women participation in SILC activities and its effects on malaria prevention and control and the role of government and NGOs in general social and economic and malaria-related activities. Further collaboration between governmental and NGOs is crucial in improving the financial base of women and enhancing participation of community health workers in malaria control and prevention if we are to achieve the goal of malaria elimination.
